# Changes in Food Insecurity Among US Adults With Low Income During the COVID-19 Pandemic

**DOI:** 10.1001/jamanetworkopen.2024.62277

**Published:** 2025-02-28

**Authors:** Yingfei Wu, Jessica Cheng, Anne N. Thorndike

**Affiliations:** 1 Division of General Internal Medicine, Massachusetts General Hospital, Boston; 2 Harvard Medical School, Boston, Massachusetts; 3 Department of Epidemiology, Harvard T.H. Chan School of Public Health, Boston, Massachusetts

## Abstract

**Importance:**

Racial and ethnic minority groups disproportionately experience food insecurity. During the COVID-19 pandemic, the US enacted temporary food assistance policies, including emergency allotments for Supplemental Nutrition Assistance Program (SNAP) benefits. The effects of the pandemic and these policies on food insecurity by race and ethnicity are unclear.

**Objective:**

To examine prevalence trends in food insecurity by racial and ethnic groups and SNAP use before and during the pandemic.

**Design, Setting, and Participants:**

This cross-sectional survey study analyzed National Health Interview Survey data before (January 2019 to March 2020) and during the COVID-19 pandemic (April 2020 to December 2022). Participants were noninstitutionalized US adults (≥18 years) with low income (<200% federal poverty level). Statistical analysis was performed from September 25, 2023, to February 27, 2024.

**Exposure:**

COVID-19 pandemic time period.

**Main Outcomes and Measures:**

Food insecurity was measured using the 10-item US Department of Agriculture Adult Food Security Survey module, categorizing participants as food secure (high or marginal food security) or insecure (low or very low food security). Survey-weighted Poisson regressions were modeled to examine changes in food insecurity prevalence over time by race and ethnicity (Asian, Black, Hispanic, and White) and by race and ethnicity and SNAP use (yes or no) including a 3-way interaction term (time × race and ethnicity × SNAP).

**Results:**

Among 30 396 adults with low income, approximately one-half were female (56.0% [95% CI, 54.7%-57.2%] during the pre–COVID-19 time period; 57.4% [95% CI, 56.4%-58.4%] during the COVID-19 time period). Food insecurity prevalence decreased from 20.9% (95% CI, 19.9%-22.0%) before the COVID-19 pandemic to 18.8% (95% CI, 17.9%-19.7%) during the pandemic (*P* < .001). SNAP use prevalence increased overall (from 31.5% [95% CI, 30.1%-32.9%] to 36.0% [95% CI, 34.8%-37.3%]; *P* < .001) and for each racial and ethnic group. There were no significant differences in food insecurity changes over time by racial and ethnic group (Wald test *F* = 1.29; *P* = .28 for 2-way interaction). Among SNAP participants, food insecurity decreased for Asian, Hispanic, and White adults but did not change for Black adults; among non-SNAP participants, food insecurity did not change for Black, Hispanic, and White adults but increased for Asian adults (Wald test *F* = 4.43; *P* = .02 for 3-way interaction).

**Conclusions and Relevance:**

During the COVID-19 pandemic, food insecurity decreased among SNAP participants in most racial and ethnic groups but did not decrease among non-SNAP participants in any group. These results suggest that during the pandemic, increased SNAP benefit amounts were associated with ameliorating food insecurity for many US adults who were able to access SNAP but did not reduce racial and ethnic disparities in food insecurity.

## Key Points

**Question:**

During the COVID-19 pandemic when Supplemental Nutrition Assistance Program (SNAP) benefit amounts were increased, how did food insecurity prevalence change among US adults with low income, and were race, ethnicity, and SNAP use associated with these changes?

**Findings:**

In this cross-sectional survey study of 30 396 adults from the National Health Interview Survey, food insecurity prevalence decreased among SNAP participants in most racial and ethnic groups but did not decrease among non-SNAP participants in any group.

**Meaning:**

These findings suggest that SNAP benefit amount increases during the pandemic were associated with ameliorating food insecurity for many adults who were able to access SNAP, but they did not reduce racial and ethnic disparities.

## Teaser

This cross-sectional survey study examines prevalence trends in food insecurity by racial and ethnic groups and Supplemental Nutrition Assistance Program use before and during the COVID-19 pandemic.

## Introduction

There have been long-standing racial and ethnic disparities in food insecurity in the US.^1,2,3^ Before the COVID-19 pandemic in 2019, 23.0% of Black and 17.0% of Hispanic households were food insecure compared with 10.7% of White households.^4^ Given the association between food insecurity and diet-related chronic diseases, such as obesity and diabetes, racial and ethnic disparities in these health conditions have also been attributed to disparities in food insecurity.^5,6,7,8,9^ The Supplemental Nutrition Assistance Program (SNAP), a federal aid program for eligible low-income US households, has been shown to ameliorate food insecurity and may help address related racial and ethnic disparities.^10,11,12,13,14,15,16^ Although research early in the COVID-19 pandemic highlighted continued food insecurity differences among racial and ethnic groups,^17,18,19,20^ less is known about how food insecurity changed by race and ethnicity during the full pandemic period when changes in nutrition assistance policies were implemented.

In March 2020, the US federal government enacted the first of several food assistance program changes to alleviate the economic effects of the pandemic.^21^ SNAP emergency allotments authorized by the Families First Coronavirus Response Act allowed recipients to receive the maximum monthly benefit for their household size, relaxed administrative procedures (such as waiving interviews), and suspended work requirements for adults younger than 50 years to continue receiving SNAP benefits.^21^ Despite these changes, a 2024 analysis by Troy et al^22^ showed that although the prevalence of food insecurity decreased for US adults with low income in 2021 compared with 2019, it returned to prepandemic levels by 2022. Additionally, the SNAP emergency allotment program ended in March 2023, and all eligibility modifications were reversed by 2023.^23,24^

Although the US Department of Agriculture (USDA) publishes annual food insecurity data,^4^ it is unclear how pandemic era SNAP benefits affected food insecurity across racial and ethnic groups and whether such policies contributed to reducing disparities in food insecurity. The objective of this study was to quantify changes in food insecurity prevalence before and during the COVID-19 pandemic among adults with low income from different racial and ethnic groups and by SNAP use using a large, nationally representative database.

## Methods

### Survey Population

This study was exempt from Mass General Brigham institutional review board evaluation because the data used in this study are publicly available, deidentified data from the National Health Interview Survey (NHIS). The NHIS conducts annual interviews of approximately 30 000 individuals from respective households to create nationally representative samples of adult (≥18 years) noninstitutionalized civilians in the US population.^25^ The survey is conducted through in-person or telephone interviews and is available in English and Spanish. Additional details on survey design and sampling construction have been published elsewhere.^25^ In our cross-sectional survey study, public use data from the 2019, 2020, 2021, and 2022 cross-sectional survey cycles were used. For 2020, the adult partial sample that excluded the subset of participants (n = 2626) who were recalled from the 2019 survey was used, and corresponding survey weights were applied as recommended.^26^ The study sample comprised participants reporting income levels less than 200% of the federal poverty level (FPL) for their family size because 200% of the FPL is the maximum income limit that states may choose to accept for SNAP eligibility.^27^ Because many US states set the maximum income limit as less than 130% of the FPL, we conducted sensitivity analyses using a smaller sample of participants reporting less than 125% of the FPL (the closest delineation available in the NHIS). Survey years before 2019 were not included because of a redesign of the NHIS in 2019 that may have influenced prevalence estimates.^28^ Missing data made up a low percentage (<5%), and no observations were deleted. This cross-sectional survey study followed the Strengthening the Reporting of Observational Studies in Epidemiology (STROBE) reporting guideline.

### Measures

Although the pandemic began affecting some of the US population in early March 2020, states were not enrolled in SNAP emergency allotments until late March or early April.^29^ Therefore, this study used April 2020 as the cutoff point between pre–COVID-19 and COVID-19 time periods; for the 2020 survey cycle, January-March data were included in the pre–COVID-19 time period (along with all of 2019), whereas April-December data were included in the COVID-19 time period (along with all of 2021 and 2022). A total of 33 US states and the District of Columbia remained in the emergency allotment program throughout the entirety of the examined COVID-19 period (ie, through the end of 2022). Participants self-reported race and ethnicity separately, and race and ethnicity were categorized using a combined variable with non-Hispanic Asian (hereafter referred to as Asian), non-Hispanic Black or African American (hereafter referred to as Black), Hispanic, and non-Hispanic White (hereafter referred to as White) groups. Other racial and ethnic group categories, including non-Hispanic American Indian or Alaska Native only, non-Hispanic American Indian or Alaska Native and any other group, and other single and multiple races, were not assessed because of small sample sizes.

Food insecurity within the past 30 days was measured using the 10-item USDA Adult Food Security Survey module.^25^ Raw scores (range, 0-10) were dichotomized into food secure (0-2, high to marginal food security) or food insecure (3-10, low to very low food security). A household was considered to have received SNAP if participants answered “yes” to any household family member receiving food stamps in the past 12 months. Cohort characteristics included age, sex, body mass index (calculated as weight in kilograms divided by height in meters squared) category (<18.5, 18.5 to <25, 25 to <30, or ≥30), Special Supplemental Nutrition Program for Women, Infants, and Children use in the past 12 months (yes or no), working in the past week (yes or no), health insurance (yes or no), and education level (less than high school, high school or equivalent, or more than high school).

### Statistical Analysis

Statistical analysis was performed from September 25, 2023, to February 27, 2024. Cohort characteristics were reported in sample count data and weighted survey proportions and compared during the pre–COVID-19 and COVID-19 periods using *t* tests for continuous variables and χ^2^ tests for categorical variables. SNAP use prevalence during the pre–COVID-19 and COVID-19 periods were compared overall and by race and ethnicity using χ^2^ tests.

To assess whether food insecurity prevalence over time differed by race and ethnicity, a Poisson regression including a 2-way interaction term (race and ethnicity × time) was modeled. To assess whether food insecurity prevalence over time differed by race and ethnicity and SNAP use, a Poisson regression including 2-way interaction terms (race and ethnicity × time, SNAP × time, and SNAP × race and ethnicity) and a 3-way interaction term (race and ethnicity × SNAP × time) was modeled, with the 3-way interaction being the effect of interest. Poisson regressions were used to estimate prevalence ratios, were survey weighted, used linearized robust variance estimation, and were not adjusted for any covariates.

Crude prevalence estimates before and during the COVID-19 pandemic were presented for each racial and ethnic group and for racial and ethnic group by SNAP use category, as well as for their respective prevalence ratios and *P* values, using linear combinations of coefficients. The significance of main effects and interaction terms were evaluated using postestimation Wald tests. Results were considered statistically significant if 2-tailed *P* < .05, and results were not adjusted for multiple comparison. All analyses were conducted with corresponding survey weights (this involved creating a combined weight including the full survey weights [wtfa_a] for 2019, 2021, and 2022 and the partial survey weight [wtsa_p] for 2020)^26^ and strata ([pstrat]) using the svy: command with the subpop() option as applicable in Stata, version 16.1 (StataCorp LLC).

## Results

### Cohort Characteristics

A total of 30 396 participants were analyzed, corresponding to a representative US population of 66 860 158. Characteristics of the pre–COVID-19 and COVID-19 cohorts are presented in Table 1. Approximately one-half were female (56.0% [95% CI, 54.7%-57.2%] during the pre–COVID-19 time period; 57.4% [95% CI, 56.4%-58.4%] during the COVID-19 time period), and approximately one-half reported White race (47.0% [95% CI, 44.8%-49.2%] during the pre–COVID-19 time period; 45.4% [95% CI, 43.2%-47.6%] during the COVID-19 time period). Comparing the pre–COVID-19 cohort with the COVID-19 cohort, the overall prevalence of food insecurity decreased (from 20.9% [95% CI, 19.9%-22.0%] to 18.8% [95% CI, 17.9%-19.7%]; *P* = .001), and SNAP use increased (from 31.5% [95% CI, 30.1%-32.9%] to 36.0% [95% CI, 34.8%-37.3%]; *P* < .001). SNAP use increased during the COVID-19 pandemic across all examined racial and ethnic groups (eTable 1 in Supplement 1). Among SNAP users only, there were no additional differences in cohort characteristics before and during the COVID-19 pandemic (eTable 2 in Supplement 1).

**Table 1.** Characteristics of US Adults With Low Income Before and During the COVID-19 Pandemic^a^

Characteristic	Before COVID-19 period		During COVID-19 period		*P* value
	No.	Weighted % (95% CI)	No.	Weighted % (95% CI)	
Sample	11 610		18 786		
Population	23 256 383		43 603 775		
Age group, y					
18-44	4599	50.6 (49.3-52.0)	7183	50.2 (49.0-51.3)	.10
45-64	3389	28.0 (26.9-29.0)	5316	27.1 (26.3-28.0)	
≥65	3593	21.4 (20.5-22.4)	6250	22.7 (21.9-23.5)	
Female	6947	56.0 (54.7-57.2)	11 481	57.4 (56.4-58.4)	.06
Race and ethnicity					
Asian	500	5.3 (4.6-6.0)	921	5.2 (4.6-5.9)	.20
Black	1986	17.4 (15.9-19.1)	3247	18.0 (16.6-19.6)	
Hispanic	2424	26.7 (24.5-28.9)	4148	27.9 (25.5-30.4)	
White	6279	47.0 (44.8-49.2)	9835	45.4 (43.2-47.6)	
Other^b^	421	3.6 (2.6-4.8)	635	3.4 (2.5-4.6)	
BMI					
<18.5	240	2.1 (1.8-2.5)	370	2.0 (1.8-2.3)	.26
18.5 to <25	3292	29.8 (28.6-31.0)	5238	28.5 (27.6-29.4)	
25 to <30	3570	31.7 (30.6-32.8)	5872	31.9 (31.0-32.8)	
≥30	4175	36.4 (35.3-37.6)	6738	37.5 (36.5-38.6)	
Food insecurity, past 30 d	2346	20.9 (19.9-22.0)	3158	18.8 (17.9-19.7)	.001
SNAP, past 12 mo	3451	31.5 (30.1-32.9)	6182	36.0 (34.8-37.3)	<.001
WIC, past 12 mo	799	15.8 (14.5-17.2)	1239	16.2 (15.2-17.4)	.57
Worked past week	4737	48.8 (47.5-50.0)	6965	45.4 (44.4-46.5)	<.001
Health insurance	9648	78.6 (77.3-79.9)	15 998	80.5 (79.2-81.8)	.004
Education level					
Less than high school	2483	26.8 (25.5-28.2)	3848	24.1 (23.0-25.2)	<.001
High school or equivalent	4057	34.5 (33.2-35.8)	6841	38.2 (37.1-39.2)	
More than high school	4962	38.7 (37.4-40.0)	7950	37.8 (36.6-38.9)	

Abbreviations: BMI, body mass index (calculated as weight in kilograms divided by height in meters squared); SNAP, Supplemental Nutrition Assistance Program; WIC, Special Supplemental Nutrition Program for Women, Infants, and Children.

^a^ Missing data during the pre–COVD-19 period (January 2019 to March 2020): age (n = 29), BMI category (n = 333), SNAP (n = 437), WIC (n = 6251), food insecurity (n = 390), worked past week (n = 337), health insurance (n = 42), children in household (n = 45), and education level (n = 108); missing data during the COVID-19 period (April 2020 to December 2022): age (n = 37), BMI category (n = 568), SNAP (n = 1038), WIC (n = 10 528), food insecurity (n = 958), worked past week (n = 846), health insurance (n = 76), and education level (n = 147).

^b^ Includes non-Hispanic American Indian or Alaska Native only, non-Hispanic American Indian or Alaska Native and any other group, and other single and multiple races.

### Food Insecurity by Race and Ethnicity

During the pre–COVID-19 period, food insecurity prevalence was 10.9% (95% CI, 7.5%-14.3%) in Asian adults, 26.0% (95% CI, 23.4%-28.7%) in Black adults, 20.2% (95% CI, 17.9%-22.5%) in Hispanic adults, and 19.5% (95% CI, 18.2%-20.7%) in White adults (Table 2). Overall, there were no significant differential changes between the pre–COVID-19 and COVID-19 periods in food insecurity prevalence by race and ethnicity (Wald test *F* = 1.29; *P* = .28 for 2-way interaction) (eTable 3 in Supplement 1). In post hoc testing, White adults had a significant decrease in food insecurity (prevalence ratio, 0.85; 95% CI, 0.78-0.93), Black and Hispanic adults had smaller decreases in food insecurity that were not statistically significant, and Asian adults had an increase in food insecurity that was not statistically significant. Results were similar among those reporting less than 125% of the FPL (eTables 4 and 5 in Supplement 1).

**Table 2.** Crude Prevalences and Crude Prevalence Ratios of Food Insecurity by Racial and Ethnic Group Among US Adults With Low Income Before and During the COVID-19 Pandemic^a^

Race and ethnicity	Food insecurity, % (95% CI)		Prevalence ratio (95% CI)	*P* value
	Before COVID-19 period	During COVID-19 period		
Asian	10.9 (7.5-14.3)	12.3 (9.6-15.0)	1.13 (0.79-1.63)	.51
Black	26.0 (23.4-28.7)	24.4 (22.5-26.4)	0.94 (0.82-1.07)	.35
Hispanic	20.2 (17.9-22.5)	19.2 (17.6-20.8)	0.95 (0.83-1.08)	.43
White	19.5 (18.2-20.7)	16.6 (15.4-17.8)	0.85 (0.78-0.93)	.001

^a^ Before the COVID-19 pandemic was from January 2019 to March 2020; during the pandemic was from April 2020 to December 2022.

### Food Insecurity by Race and Ethnicity and SNAP Use

The Figure shows prevalence estimates for SNAP and non-SNAP participants by racial and ethnic group. There were differential changes in food insecurity prevalence over time by race and ethnicity and SNAP use (Wald test *F* = 3.45; *P* = .02 for 3-way interaction) (eTable 6 in Supplement 1). Among SNAP participants, food insecurity decreased in Asian (from 31.0% [95% CI, 19.1%-43.0%] to 16.3% [95% CI, 10.6%-22.0%]; *P* = .02), Hispanic (from 32.6% [95% CI, 28.1%-37.1%] to 22.8% [95% CI, 20.2%-25.4%]; *P* < .001), and White (from 37.0% [95% CI, 34.0%-40.0%] to 27.6% [95% CI, 25.2%-30.0%]; *P* < .001) adults but did not change significantly in Black adults (from 33.5% [95% CI, 28.9%-38.1%] to 28.2% [95% CI, 25.0%-31.5%]; *P* = .06) (Figure; eTable 7 in Supplement 1). Among non-SNAP participants, food insecurity did not change significantly among Black, Hispanic, and White adults but increased in Asian adults (from 6.2% [95% CI, 3.4%-8.9%] to 10.7% [95% CI, 7.4%-14.0%]; *P* = .04; Wald test *F* = 4.43; *P* = .02 for 3-way interaction). Results were similar among those reporting less than 125% of the FPL (eTables 8 and 9 in Supplement 1).

**Figure.**
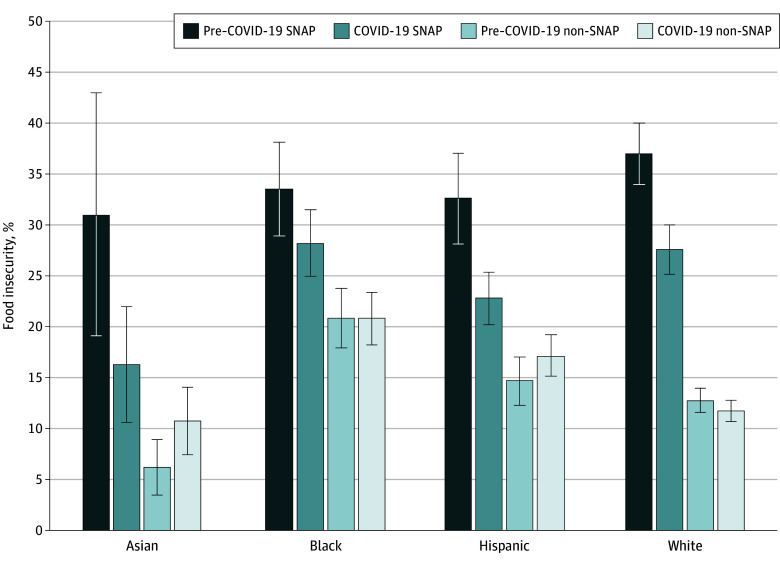
Food Insecurity Before and During the COVID-19 Pandemic for US Adults With Low Income, by Race and Ethnicity and SNAP Use Pre–COVID-19 was from January 2019 to March 2020; COVID-19 period was from April 2020 to December 2022. SNAP indicates Supplemental Nutrition Assistance Program.

## Discussion

This study found that food insecurity prevalence decreased during the pandemic compared with the prepandemic period for US White adults with low income, but it did not change significantly for Hispanic, Asian, and Black adults. There were differential associations of both SNAP use and race and ethnicity with food insecurity changes during the COVID-19 pandemic. Food insecurity prevalence decreased during the pandemic for SNAP participants in Asian, Hispanic, and White groups but did not decrease among non-SNAP participants in any racial and ethnic group. These findings suggest that expanded SNAP benefits during the pandemic may have played an important role in decreasing food insecurity for many adults with low income who were able to access SNAP but did not appear to reduce racial and ethnic disparities in food insecurity.

Several changes to SNAP, the majority of whose beneficiaries come from racial and ethnic minority groups,^30^ were enacted throughout the examined COVID-19 period. SNAP emergency allotments, which enabled all beneficiaries to receive the maximum benefit amount for their household size, became available at the beginning of the pandemic with all US states opting to participate.^23^ In October 2021, the Thrifty Food Plan reevaluation resulted in baseline increases for all SNAP beneficiaries, along with the annual cost-of-living adjustment.^31^ SNAP eligibility, enrollment, and maintenance requirements were also broadened, including waiving interview requirements in March 2020, suspending work requirements in April 2020, extending certification periods in April 2020, and expanding eligibility for qualifying students in February 2021.^24,32,33,34^ These changes may have contributed to our findings, wherein SNAP participants from Asian, Hispanic, and White groups experienced significant decreases in food insecurity during the COVID-19 pandemic. Unfortunately, several states gradually withdrew from the emergency allotment program, and the program ended in March 2023 for the remaining 32 states and Washington, DC.^23^ Additionally, all eligibility moderations ended by October 2023.^35^

Black adults with low income continued to experience high food insecurity prevalence during the pandemic, highlighting the persistence of disparities in food insecurity among racial and ethnic minority groups during this time. Black adults have historically experienced the highest poverty rate among Asian, Black, Hispanic, and White groups in the US.^36^ During the pandemic, the national poverty rate of Black households increased from 18.8% in 2019 to 19.5% in 2020 and remained at this level in 2021 before decreasing in 2022.^36,37,38,39^ The increased poverty rates during the COVID-19 pandemic appeared consistent with increased SNAP participation for Black adults with low income from this analysis, yet there were no significant decreases in food insecurity irrespective of SNAP use. Our results are congruent with a 2024 analysis by Austin and Sokol^40^ that showed decreases in food hardship among SNAP-participating US households with White and Hispanic children but not Black children during the emergency allotment period. Many underlying factors may be associated with food insecurity for Black adults with low income, and nutrition assistance participation alone may not be able to meet the needs of this group. For example, structural racism and discrimination may be associated with long-standing disparities in food insecurity for minoritized groups^41,42,43^ and were likely exacerbated during the pandemic, including fear of going out to buy food, lack of transportation access to purchase food, and decreased ability to afford food.^44,45,46^ Some research also indicates lower enrollment in nutrition assistance programs, possibly because of lack of knowledge and exposure, as 1 study showed that two-thirds of SNAP-eligible Black adults perceived they were ineligible for the program.^47^

Our study also demonstrated a concerning food insecurity trend for the low-income Asian group. Although the sample size was smaller for the Asian group, there was evidence of higher food insecurity despite increased SNAP use during the pandemic. With more than 10 million estimated undocumented immigrants residing in the US, most of whom are of Hispanic ethnicity or Asian race,^48^ it is likely a portion of these groups would meet criteria for SNAP and other government financial assistance programs but are not eligible to enroll. Additionally, considering the vast heterogeneity of Asian populations in the US, it is easy to overlook specific groups that experience disproportionate food insecurity.^49^ For example, California state survey data have shown high food insecurity in certain Asian subpopulations, such as Vietnamese and Filipino groups, and risk of food insecurity was related to low levels of acculturation and non-English primary language use.^50,51^ As the fastest growing racial and ethnic group in the US,^52^ Asian populations warrant further evaluation into the underlying causes for and barriers to addressing food insecurity.

### Strengths and Limitations

A strength of our analysis is the use of a large, nationally representative dataset spanning from the prepandemic period in 2019 until the end of 2022 to evaluate trends by race and ethnicity, complementing earlier studies on food insecurity differences during the beginning of the pandemic.^17,18,19^

This study has some limitations. One limitation is the cross-sectional nature of the survey data that restricts inferences on causality between pandemic-era policies and food insecurity changes. The US government also enacted non-SNAP or food-related financial assistance policies, such as Economic Impact Payments and expanded unemployment benefits, that may have contributed to reduced household food insecurity.^53,54,55,56,57^ Another limitation is grouping pandemic-era data from April 2020 to December 2022; however, as the US government enacted various nutrition assistance and general financial assistance policies throughout the pandemic without well-defined periods,^23,31,53^ we chose to analyze the pandemic period as a whole. Additionally, sample sizes for Asian, Black, and Hispanic adults with low income were smaller than for White adults, which may limit the power to detect significant differences in post hoc testing. Considering that self-reported SNAP use is generally underreported in survey data,^58^ differences between SNAP and non-SNAP users in food insecurity prevalence over time may be inflated. Also, limited NHIS language options precluded participation from households with only non-English language preference members, which may bias findings for racial and ethnic minority groups.

## Conclusions

Among US adults with low income, the prevalence of food insecurity decreased during the COVID-19 pandemic compared with before the pandemic. This cross-sectional survey study found that observed decreases in food insecurity were associated with decreases in those receiving SNAP. These results suggest that pandemic-era increases in SNAP benefit amounts may have ameliorated food insecurity for some US adults with low income with access to SNAP during the COVID-19 pandemic. These findings raise concern that the abrupt end of SNAP emergency allotments and other expansion benefits may result in increases in food insecurity in the coming years. As racial and ethnic minority groups continue to have high rates of food insecurity, more research and policies are needed to improve overall food security and narrow disparities for food insecurity and related health conditions in the US.

## Notes

Supplement 1. **eTable 1.** SNAP Use in the Past 12 Months Among U.S. Adults With Low Income by Racial and Ethnic Group **eTable 2.** Characteristics of U.S. Adult SNAP Users With Low Income Before and During the COVID-19 Pandemic **eTable 3.** Wald Tests of Main Effects and Interactions Between Race/Ethnicity and Time on Food Insecurity Prevalence in U.S. Adults With Low Income Before and During the COVID-19 Pandemic **eTable 4.** Crude Prevalences and Crude Prevalence Ratios of Food Insecurity by Racial and Ethnic Group in U.S. Adults Reporting <125% of Federal Poverty Level Before and During the COVID-19 Pandemic **eTable 5.** Wald Tests of Main Effects and Interactions Between Race/Ethnicity and Time on Food Insecurity Prevalence in U.S. Adults Reporting <125% of Federal Poverty Level Before and During the COVID-19 Pandemic **eTable 6.** Wald Tests of Main Effects and Interactions Between SNAP Use, Race/Ethnicity, and Time on Food Insecurity Prevalence in U.S. Adults With Low Income Before and During the COVID-19 Pandemic **eTable 7.** Crude Prevalences and Crude Prevalence Ratios of Food Insecurity by Racial and Ethnic Group and SNAP Use in U.S. Adults With Low Income Before and During the COVID-19 Pandemic **eTable 8.** Crude Prevalences and Crude Prevalence Ratios of Food Insecurity by Racial and Ethnic Group and SNAP Use in U.S. Adults Reporting <125% of Federal Poverty Level Before and During the COVID-19 Pandemic **eTable 9.** Wald Tests of Main Effects and Interactions Between SNAP Use, Race/Ethnicity, and Time on Food Insecurity Prevalence in U.S. Adults Reporting <125% of Federal Poverty Level Before and During the COVID-19 Pandemic

Supplement 2.
Data Sharing Statement
