# Supplementary material for: Changes in Food Insecurity Among US Adults With Low Income During the COVID-19 Pandemic
Source: JAMA Netw Open. 2025 Feb 28;8(2):e2462277. doi: 10.1001/jamanetworkopen.2024.62277 (PMC11871537; doi:10.1001/jamanetworkopen.2024.62277)
Supplement: Supplement 1. — eTable 1. SNAP Use in the Past 12 Months Among U.S. Adults With Low Income by Racial and Ethnic Group eTable 2. Characteristics of U.S. Adult SNAP Users With Low Income Before and During the COVID-19 Pandemic eTable 3. Wald Tests of Main Effects and Interactions Between Race/Ethnicity and Time on Food Insecurity Prevalence in U.S. Adults With Low Income Before and During the COVID-19 Pandemic eTable 4. Crude Prevalences and Crude Prevalence Ratios of Food Insecurity by Racial and Ethnic Group in U.S. Adults Reporting <125% of Federal Poverty Level Before and During the COVID-19 Pandemic eTable 5. Wald Tests of Main Effects and Interactions Between Race/Ethnicity and Time on Food Insecurity Prevalence in U.S. Adults Reporting <125% of Federal Poverty Level Before and During the COVID-19 Pandemic eTable 6. Wald Tests of Main Effects and Interactions Between SNAP Use, Race/Ethnicity, and Time on Food Insecurity Prevalence in U.S. Adults With Low Income Before and During the COVID-19 Pandemic eTable 7. Crude Prevalences and Crude Prevalence Ratios of Food Insecurity by Racial and Ethnic Group and SNAP Use in U.S. Adults With Low Income Before and During the COVID-19 Pandemic eTable 8. Crude Prevalences and Crude Prevalence Ratios of Food Insecurity by Racial and Ethnic Group and SNAP Use in U.S. Adults Reporting <125% of Federal Poverty Level Before and During the COVID-19 Pandemic eTable 9. Wald Tests of Main Effects and Interactions Between SNAP Use, Race/Ethnicity, and Time on Food Insecurity Prevalence in U.S. Adults Reporting <125% of Federal Poverty Level Before and During the COVID-19 Pandemic [file jamanetwopen-e2462277-s001.pdf]

Open Access Specialty Journal Articles with CC-BY License

Supplemental Online Content

Wu Y, Cheng J, Thorndike AN. Changes in food insecurity among US adults with low income during the COVID-19 pandemic. *JAMA Netw Open*. 2025;8(2):e2462277. doi:10.1001/jamanetworkopen.2024.62277

SUPPLEMENTAL ONLINE CONTENT

eTable 1. SNAP Use in the Past 12 Months Among U.S. Adults With Low Income by Racial and Ethnic Group

eTable 2. Characteristics of U.S. Adult SNAP Users With Low Income Before and During the COVID-19 Pandemic

eTable 3. Wald Tests of Main Effects and Interactions Between Race/Ethnicity and Time on Food Insecurity Prevalence in U.S. Adults With Low Income Before and During the COVID-19 Pandemic

eTable 4. Crude Prevalences and Crude Prevalence Ratios of Food Insecurity by Racial and Ethnic Group in U.S. Adults Reporting <125% of Federal Poverty Level Before and During the COVID-19 Pandemic

eTable 5. Wald Tests of Main Effects and Interactions Between Race/Ethnicity and Time on Food Insecurity Prevalence in U.S. Adults Reporting <125% of Federal Poverty Level Before and During the COVID-19 Pandemic

eTable 6. Wald Tests of Main Effects and Interactions Between SNAP Use, Race/Ethnicity, and Time on Food Insecurity Prevalence in U.S. Adults With Low Income Before and During the COVID-19 Pandemic

eTable 7. Crude Prevalences and Crude Prevalence Ratios of Food Insecurity by Racial and Ethnic Group and SNAP Use in U.S. Adults With Low Income Before and During the COVID-19 Pandemic

eTable 8. Crude Prevalences and Crude Prevalence Ratios of Food Insecurity by Racial and Ethnic Group and SNAP Use in U.S. Adults Reporting <125% of Federal Poverty Level Before and During the COVID-19 Pandemic

eTable 9. Wald Tests of Main Effects and Interactions Between SNAP Use, Race/Ethnicity, and Time on Food Insecurity Prevalence in U.S. Adults Reporting <125% of Federal Poverty Level Before and During the COVID-19 Pandemic

This supplemental material has been provided by the authors to give readers additional information about their work.

© 2025 Wu Y et al. *JAMA Network Open*.

eTable 1. SNAP use in the past 12 months among U.S. adults with low income by racial and ethnic group*

	SNAP past 12 mo, % (95% CI)		
	**Before COVID-19 period**	**During COVID-19 period**	***P* value**
**Asian**	19.6 (15.2-24.7)	28.8 (24.7-33.3)	0.004
**Black**	43.2 (40.1-46.4)	49.6 (47.1-52.2)	0.001
**Hispanic**	30.7 (28.3-33.1)	35.7 (33.2-38.2)	0.001
**White**	27.8 (26.2-29.5)	31.1 (29.6-32.7)	0.001

* SNAP- Supplemental Nutrition Assistance Program; Pre-COVID: January 2019- March 2020; COVID: April 2020- December 2022

© 2025 Wu Y et al. *JAMA Network Open*.

eTable 2. Characteristics of U.S. adult SNAP users with low income before and during the COVID-19 pandemic*

	Before COVID-19 period		During COVID-19 period		*P* Value
	No.	Weighted % (95% CI)	No.	Weighted % (95% CI)	
**Sample**	3451		6182		
**Population**	7 017 789		16 277 031		
**Age group, years**					
**18-44**	1509	53.6 (51.4-55.8)	2688	54.3 (52.6-56.1)	
**45-64**	1173	30.4 (28.4-32.3)	2022	29.7 (28.2-31.3)	
**≥65**	767	16.0 (14.7-17.5)	1466	16.0 (14.9-17.1)	0.83
**Female**	2295	62.8 (60.6-64.9)	4088	62.0 (60.3-63.7)	0.58
**Race/Ethnicity**					
**Asian**	85	3.3 (2.4-4.3)	242	4.2 (3.5-5.1)	
**Black**	804	23.6 (23.0-29.2)	1457	24.3 (22.0-26.8)	
**Hispanic**	754	26.0 (23.0-29.2)	1440	27.4 (24.3-30.7)	
**White**	1630	41.7 (38.8-44.8)	2772	39.7 (36.8-42.5)	
**Other**	178	5.4 (3.5-7.8)	271	4.4 (2.9-6.3)	0.16
**BMI Category, kg/m2**					
**<18.5**	70	2.1 (1.5-2.8)	92	1.6 (1.2-2.0)	
**18.5 to <25**	844	25.9 (23.9-27.9)	1513	25.6 (24.0-27.1)	
**25 to <30**	964	29.3 (27.2-31.4)	1809	29.5 (28.0-31.0)	
**≥30**	1485	42.8 (40.5-45.1)	2618	43.4 (41.6-45.1)	0.61
**Food Insecurity Past 30d**	1244	35.2 (33.0-37.4)	1563	26.1 (24.5-27.8)	<0.001
**WIC Past 12mo**	478	26.3 (23.7-29.1)	795	25.2 (23.2-27.2)	0.47
**Worked Past Week**	1140	38.1 (36.0-40.2)	2054	39.0 (37.3-40.7)	0.49
**Health Insurance**	2940	81.4 (79.2-83.4)	5442	83.6 (81.7-85.4)	0.05
**Education Level**					
**Less Than High School**	973	34.9 (32.5-37.2)	1611	29.7 (27.8-31.6)	
**High School or Equivalent**	1199	33.7 (31.7-35.8)	2191	38.4 (36.7-40.1)	
**More Than High School**	1255	31.4 (29.3-33.6)	2323	32.0 (30.3-33.6)	<0.001

* Pre-COVID: January 2019- March 2020; COVID: April 2020- December 2022; SNAP- Supplemental Nutrition Assistance Program

© 2025 Wu Y et al. *JAMA Network Open*.

eTable 3. Wald tests of main effects and interactions between Race/Ethnicity and Time on food insecurity prevalence in U.S. adults with low income before and during the COVID-19 pandemic*

	**F Value**	***P* Value**
**Race/Ethnicity**	13.40	<0.001
**Time**	12.04	<0.001
**Race/Ethnicity*Time**	1.29	0.28

* Pre-COVID: January 2019- March 2020; COVID: April 2020- December 2022

© 2025 Wu Y et al. *JAMA Network Open*.

eTable 4. Crude prevalences and crude prevalence ratios of food insecurity by racial and ethnic group in U.S. adults reporting <125% of federal poverty level before and during the COVID-19 pandemic*

	Food Insecurity Before COVID-19 Period % (95% CI)	Food Insecurity During COVID-19 Period % (95% CI)	PR† (95% CI)	*P* Value
**Asian**	14.3 (9.8-18.7)	16.0 (11.5-20.4)	1.12 (0.75-1.67)	0.57
**Black**	30.6 (26.9-34.3)	28.2 (25.6-30.7)	0.92 (0.79-1.07)	0.29
**Hispanic**	26.6 (23.3-29.8)	23.9 (21.7-26.2)	0.90 (0.78-1.04)	0.15
**White**	25.7 (23.6-27.8)	21.7 (19.8-23.6)	0.84 (0.75-0.94)	0.003

* Pre-COVID: January 2019- March 2020; COVID: April 2020- December 2022
† PR- Prevalence Ratio

© 2025 Wu Y et al. *JAMA Network Open*.

eTable 5. Wald tests of main effects and interactions between Race/Ethnicity and Time on food insecurity prevalence in U.S. adults reporting <125% of federal poverty level before and during the COVID-19 pandemic*

	F Value	*P* Value
**Race/Ethnicity**	6.80	<0.001
**Time**	8.98	0.003
**Race/Ethnicity*Time**	0.84	0.47

* Pre-COVID: January 2019- March 2020; COVID: April 2020- December 2022

© 2025 Wu Y et al. *JAMA Network Open*.

eTable 6. Wald tests of main effects and interactions between SNAP use, Race/Ethnicity, and Time on food insecurity prevalence in U.S. adults with low income before and during the COVID-19 pandemic*

	**F Value**	***P* Value**
**SNAP**	320.11	<0.001
**Race/Ethnicity**	15.80	<0.001
**Time**	1.75	0.19
**SNAP*Time**	6.85	0.009
**SNAP*Race/Ethnicity**	10.42	<0.001
**Race/Ethnicity*Time**	2.74	0.04
**Race/Ethnicity*SNAP*Time**	3.43	0.02

* SNAP- Supplemental Nutrition Assistance Program; Pre-COVID: January 2019- March 2020; COVID: April 2020- December 2022

© 2025 Wu Y et al. *JAMA Network Open*.

eTable 7. Crude prevalences and crude prevalence ratios of food insecurity by racial and ethnic group and SNAP use in U.S. adults with low income before and during the COVID-19 pandemic*

		Food Insecurity Before COVID-19 Period % (95% CI)	Food Insecurity During COVID-19 Period % (95% CI)	PR[†] (95% CI)	*P* Value
**Asian**	**SNAP**	31.0 (19.1-43.0)	16.3 (10.6-22.0)	0.53 (0.31-0.89)	0.02
	**No SNAP**	6.2 (3.4-8.9)	10.7 (7.4-14.0)	1.74 (1.02-2.98)	0.04
**Black**	**SNAP**	33.5 (28.9-38.1)	28.2 (25.0-31.5)	0.84 (0.71-1.01)	0.06
	**No SNAP**	20.8 (17.9-23.8)	20.8 (18.2-23.4)	1.00 (0.83-1.21)	0.99
**Hispanic**	**SNAP**	32.6 (28.1-37.1)	22.8 (20.2-25.4)	0.70 (0.59-0.83)	<0.001
	**No SNAP**	14.7 (12.3-17.0)	17.1 (15.1-19.2)	1.17 (0.97-1.41)	0.11
**White**	**SNAP**	37.0 (34.0-40.0)	27.6 (25.2-30.0)	0.75 (0.67-0.83)	<0.001
	**No SNAP**	12.7 (11.6-13.9)	11.7 (10.7-12.8)	0.92 (0.81-1.04)	0.19

* SNAP- Supplemental Nutrition Assistance Program; Pre-COVID: January 2019- March 2020; COVID: April 2020- December 2022
† PR- Prevalence Ratio

© 2025 Wu Y et al. *JAMA Network Open*.

eTable 8. Crude prevalences and crude prevalence ratios of food insecurity by racial and ethnic group and SNAP use in U.S. adults reporting <125% of federal poverty level before and during the COVID-19 pandemic*

		**Food Insecurity Before COVID-19 Period % (95% CI)**	**Food Insecurity During COVID-19 Period % (95% CI)**	**PR† (95% CI)**	***P* Value**
**Asian**	**SNAP**	38.7 (25.8-51.6)	18.7 (11.7-25.7)	0.48 (0.30-0.79)	0.003
	**No SNAP**	6.7 (3.3-20.0)	14.2 (7.9-20.4)	2.12 (1.09-4.11)	0.03
**Black**	**SNAP**	35.6 (30.4-40.8)	30.0 (26.3-33.7)	0.84 (0.69-1.03)	0.09
	**No SNAP**	24.9 (20.1-29.7)	25.3 (21.3-29.3)	1.02 (0.79-1.31)	0.91
**Hispanic**	**SNAP**	35.6 (30.4-40.8)	24.5 (21.3-27.8)	0.69 (0.57-0.83)	<0.001
	**No SNAP**	19.6 (15.9-23.2)	23.3 (20.0-26.7)	1.19 (0.95-1.49)	0.12
**White**	**SNAP**	39.9 (36.5-43.3)	30.3 (27.2-33.3)	0.76 (0.67-0.86)	<0.001
	**No SNAP**	15.7 (13.5-17.8)	14.2 (12.3-16.1)	0.91 (0.74-1.10)	0.33

* SNAP- Supplemental Nutrition Assistance Program; Pre-COVID: January 2019- March 2020; COVID: April 2020- December 2022
† PR- Prevalence Ratio

© 2025 Wu Y et al. *JAMA Network Open*.

eTable 9. Wald tests of main effects and interactions between SNAP use, Race/Ethnicity, and Time on food insecurity prevalence in U.S. adults reporting <125% of federal poverty level before and during the COVID-19 pandemic*

	F Value	*P* Value
**SNAP**	124.13	<0.001
**Race/Ethnicity**	10.27	<0.001
**Time**	0.95	0.33
**SNAP*Time**	2.33	0.13
**SNAP*Race/Ethnicity**	9.97	<0.001
**Race/Ethnicity*Time**	2.62	0.05
**Race/Ethnicity*SNAP*Time**	3.98	<0.001

* SNAP- Supplemental Nutrition Assistance Program; Pre-COVID: January 2019- March 2020; COVID: April 2020- December 2022

© 2025 Wu Y et al. *JAMA Network Open*.
