# Supplementary material for: Changes in Food Insecurity Among US Adults With Low Income During the COVID-19 Pandemic
Source: JAMA Netw Open. 2025 Feb 28;8(2):e2462277. doi: 10.1001/jamanetworkopen.2024.62277 (PMC11871537; doi:10.1001/jamanetworkopen.2024.62277)
Supplement: Supplement 2. — Data Sharing Statement [file jamanetwopen-e2462277-s002.pdf]

Data Sharing Statement

Wu. Changes in Food Insecurity Among US Adults With Low Income During the COVID-19 Pandemic. *JAMA Netw Open*. Published February 28, 2025.
doi:10.1001/jamanetworkopen.2024.62277

Data
Data available: Yes
Data types: Deidentified participant data
How to access data: Authors used publicly available data from the National Health Interview Survey.
When available: With publication

Supporting Documents
Document types: Statistical/analytic code
How to access documents: Statistical code may be made available upon request to yiwu1@mgh.harvard.edu
When available: With publication

Additional Information
Who can access the data: Statistical code may be made available upon request to any requester
Types of analyses: Statistical code may be made available upon request for any purpose
Mechanisms of data availability: Statistical code will be sent via email upon request
